# Birth preparedness and related factors: a cross-sectional study in Tanzania City area

**DOI:** 10.1186/s12913-021-06853-y

**Published:** 2021-08-14

**Authors:** Yoko Shimpuku, Beatrice Mwilike, Keiko Ito, Dorkasi Mwakawanga, Naoki Hirose, Kazumi Kubota

**Affiliations:** 1 Graduate School of Biomedical and Health Sciences, Hiroshima University, 1-2-3 Kasumi, Minami-ku, 730-0045 Hiroshima, Japan; 2 School of Nursing, Muhimbili University of Health and Allied Sciences, P. O. Box 65001, Dar es Salaam, Tanzania; 3 Kyoto University Hospital, 53 Shogoin-kawaharacho, Sakyo-ku, 606-8507 Kyoto, Japan; 4 Department of Biostatistics, Yokohama City University School of Medicine, 3-9 Fukuura, Kanazawa-ku, 236-0004 Yokohama, Japan

**Keywords:** Birth preparedness, Antenatal care, Pregnancy, Baseline survey, Tanzania

## Abstract

**Background:**

Birth preparedness could be the key factor that influences the choice of birthplace with skilled birth attendants. To reduce the high maternal mortality of Tanzania, a large study was planned to develop a smartphone app to promote birth preparedness in a city area of Tanzania. This study aimed to identify factors that influence birth preparedness in the city area of Tanzania.

**Methods:**

Pregnant women were asked to complete the Birth Preparedness Questionnaire during antenatal visits using tablets. Multiple linear regression analyses were performed to determine the sociodemographic and obstetric characteristics that influenced the factors.

**Results:**

A total of 211 participants were included in the analysis. Distance from the nearest health facility negatively influenced the total score of the Birth Preparedness Assessment (β= 0.7, *p* = 0.02). Education higher than college positively influenced the total score (β = 4.76, *p* = 0.01). Decision-making of birthplace by other people (not women) negatively influenced *Family Support* (β=1.18, *p* = 0.03). Having jobs negatively influenced *Preparation of Money and Food* (β=-1.02, *p* < 0.01) and positively influenced the knowledge (β = 0.75, *p* = 0.03). Being single positively influenced *Preparation of Money and Food* (β = 0.35, *p* = 0.19) and *Preference of Skilled Birth Attendants* (β = 0.42, *p* = 0.04). Experience of losing a baby negatively influenced the knowledge (β=0.80, *p* < 0.01) and *Preference of Skilled Birth Attendants* (β=0.38, *p* = 0.02).

**Conclusions:**

The findings showed an updated information on pregnant Tanzanian women living in an urban area where rapid environmental development was observed. Birth preparedness was negatively affected when women reside far from the health facilities, the birthplace decision-making was taken by others beside the women, women have jobs, and when women have experienced the loss of a baby. We hope to use the information from this study as content in our future study, in which we will be applying a smartphone app intervention for healthy pregnancy and birth preparedness. This information will also help in guiding the analysis of this future study. Although generalization of the study needs careful consideration, it is important to reconsider issues surrounding birth preparedness as women’s roles both in the family and society, are more, especially in urban settings.

## Background

In the Sustainable Development Goals #3, the target of maternal mortality ratio (MMR) is less than 70 per 100,000 live births [1]; however, Tanzania still struggles with a high MMR of 556 per 100,000 live births [2]. In countries with high maternal mortality, access to skilled birth attendants (SBAs) in health care facilities, where life-saving technology is available, is strongly recommended [3, 4]. Shimpuku, Patil, Norr, and Hill [5] reported that women who had a hospital birth in rural Tanzania were also aware of the importance of giving birth with SBAs in order to avoid complications. Although it has gradually increased, the Demographic and Health Survey (DHS) in 2015 showed that 64 % of all deliveries were conducted by SBAs, and that women who attended antenatal care (ANC) more than 4 times were 51 % [2]. In other words, nearly half of women still do not have enough access to SBAs during pregnancy and childbirth.

Researchers indicate that birth preparedness (BPR) could be the key factor that influences the choice of birthplace with SBAs [6–8]. BPR include: (a) knowledge of danger signs; (b) plan for where to give birth; (c) plan for a birth attendant; (d) plan for transportation; and (e) plan for saving money [9]. In Moshi et al.’s [10] study in a rural setting of Bukwa, BPR levels were positively influenced by age, having ever heard about BPR, being of Mambwe ethnicity, living near a health center rather than a dispensary and having had a prior preterm delivery. Another study in Chamwino District showed that significant determinants of BPR and complication readiness were found to be maternal education, spouse employment, booking at ANC, four or more antenatal visits, and knowledge of key danger signs [7]. In Urassa’s [11] study in Mpwapwa District, in the bivariate analysis, age of the woman, education, marital status, number of ANC visits, and knowing ≥ 3 obstetric danger signs were associated with BPR and complication readiness. In multivariate logistic regression analysis, women with primary education and above were twice more likely to be prepared and ready for birth and complications. Women who knew ≥ 3 obstetric danger signs were 3 times more likely to be prepared for birth and complications.

To increase BPR of pregnant women and their families, Shimpuku et al. [12] used a family-oriented group education strategy in Korogwe district and succeeded in increasing BPR of pregnant women and their family, which positively affected reducing maternal and neonatal complications. To promote this health education among local midwives, Oka, et al. [13] suggested the importance of job-aid, i.e., a tool to quickly review the essential knowledge to provide more information to pregnant women during antenatal visits. Job aids were found to be helpful for understanding and recalling information for both health providers and pregnant women [14, 15]. In recent years, job aid tools are facing digital transformation to becoming smartphone apps. It could be quite useful, especially in Tanzania, where mobile phone ownership is rapidly increasing. A large study is planned to introduce a smartphone app as a job aid to increase BPR; this will be conducted in an urban area of Tanzania because of the stable network. For the application of the “Internet of Medical Things,” the information should be real-time to address different medical scenarios [16]. However, the current evidence is limited to the rural areas of Tanzania. It is said that when capacities for measuring environmental and health indicators are limited at the national level, the public health policies will not be able to integrate environmental health indicators clearly and fully for the protection of public health [17]. Hence, it is necessary to evaluate the current state of public health indicators and related factors so that it can inform the contents of the app to be developed. To obtain information in urban area where fast development and change in women’s lives are prominent, this study aimed to identify factors that might influence BPR in urban area of Tanzania.

## Methods

### Design

This is a baseline survey to develop a smartphone app to promote BPR in a city area of Tanzania. This cross-sectional study investigated which factors might influence BPR.

### Settings

The study took place in two health centers in Dar es Salaam, the largest city in Tanzania. The population was 4,364,541, and the distributions of residential households in urban areas by region were 100 % [2]. There is one national hospital and five regional hospitals, which has the highest number of health care providers and drug availability, especially 44.7 % of medical doctors reside in Dar es Salaam while the area has 10 % of the national population [18]. In urban areas, the percentages of ANC visits of more than 4 times was 63.8 %, whereas that of rural areas was 45.0 %. Urban women are more than twice as likely as rural women to receive ANC from doctors, assistant medical officers (AMOs), clinical officers, and assistant clinical officers (19 % versus 8 %, respectively) [2]. Women in Dar es Salaam were reported as the highest percentages (35 %) in receiving ANC. The percentage of women receiving ANC from doctors, AMOs, clinical officers, and assistant clinical officers rises with a woman’s educational level, and it is similar among women in the wealth quintile, which rises from 5 % at the lowest level to 22 % at the highest wealth quintile [2].

### Samples

The inclusion criteria for women were: currently pregnant in 2nd or 3rd trimester, 16 years or older, and able to read Kiswhaili. For the baseline study, sample size was calculated for a later study to compare two groups following the intervention under preparation; therefore, the basic formula for two groups, a two-sided alternative and normal distributions with the same variances, was utilized. The sample size was calculated as 64 for each group to detect a difference (10 points) between groups at a 5 % level of significance with 80 % power. Concerning 40 % of missing data, the minimum number of participants needed was 90. Hence, at least the participants of 180 were expected for this study.

### Measurement

Information was collected on the following sociodemographic and obstetric background data: age, marital status, education, occupation, financial status, household assets, parity, distance from the nearest health center, the number of ANC visits, decision of birthplace, first antenatal visit for this pregnancy, preference of birth attendants, and for multipara women, experience of caesarean section, and loss of baby. Any identifiable information such as names or phone numbers were not collected.

In a previous study [19], the research team developed the Birth Preparedness Questionnaire (BPQ) both for pre-/post-test of an intervention study. The BPQ is a 34-item self-administered questionnaire consisting of a knowledge test and a BPR assessment. The 10-item knowledge test asked about safe pregnancy and danger signs deprived from the Integrated Management of Pregnancy and Childbirth [20]. Since the responses were “Yes/No,” and the score was calculated as 1 point per correct answer, the maximum score was 10. Another 24-item BPR assessment test is composed of items that were related to psychological values and beliefs. The items were rated using a 3-point Likert scale indicating (1) disagree, (2) neither disagree nor agree, or (3) agree, and the maximum score was 72. The factors that compose the BPR included: *Home-based value* (7 items), *BPR* (5 items), *Family support* (4 items), *Avoidance of medical intervention* (2 items), *Provision of money and food* (2 items), *Preference of SBA* (2 items), and *Pregnant women’s workload* (2 items). The higher the score, the more the person understood the recommendations of the World Health Organization (WHO) in terms of the BPR and birth attended by skilled personnel. The questionnaire was administered in Kiswahili because Kiswahili is a language familiar to most Tanzanians. The data were collected electronically using tablets.

### Data collection

The second, third, and fourth authors visited the data collection sites with assistants and mobile tablets. They approached pregnant women at the antenatal clinic and asked them to participate in the study. They explained the purpose and ethical consideration, and only those who agreed to participate completed the questionnaire.

### Data analysis

To determine the participants’ characteristics that were associated with each item (total of BPR score, knowledge score, and factors of: *Home-based value*, *BPR*, *Family support*, *Avoidance of medical intervention*, *Provision of money and food*, *Preference of SBA*, and *Pregnant women’s workload*), multiple regression analyses were conducted. The independent variables included in the multiple regression analyses were categorized as follows: marital status (single or others [married, divorced, and widowed]), education (< college or ≥ college), occupation (housewife or others (business, tailor, teacher, specialist, farmer, assistant, media, student)), financial status (≤ 5000 Tanzanian shillings [TSH] or > 5000 TSH), household asset (nothing or ≥ one), parity (continuous variable), experience of caesarean section (yes or no), loss of baby (yes or no), distance from the nearest health center (> 1 h or ≤ 1 h), the number of ANC visits (< 4 or ≥ 4), first antenatal visit for this birth (> 3 or ≤ 3), decision of birthplace (with/only others or pregnant women alone), preference of birth attendants (with health care provide or without health care provide), and age (continuous variable).

The multiple imputation method was applied to the multiple regression analyses to complement missing values. The 10 imputed datasets were applied with multivariate imputations by chained equations. The estimates from the 10 imputed datasets were combined with Rubin’s rules for combining multiply imputed data. All tests were two-tailed, and the threshold of significance was a P-value of < 0.05. All statistical analyses were two-tailed and were performed using R, Version 3.0.1 and Oracle® R Enterprise, Version 1.4.1 (Oracle, Redwood Shores, CA, USA).

### Ethical consideration

 The study was conducted based on the Declaration of Helsinki, the Ethical Guidelines for Medical and Health Research Involving Human Subjects, and the regulation of Kyoto University, including harmlessness, voluntarily, anonymity, and protection of privacy, and personal information. These principles were explained during recruitment. The research team explained the purpose, methods, and ethical considerations and ask each participant if they agree to participate in the study. Only those who agreed participated in the study. The informed consent was obtained and checked at the first page of the electronic survey form. Ethical clearance and permissions were obtained from (1) Kyoto University Graduate School and Faculty of Medicine, Ethics Committee (C1446), (2) National Institute for Medical Research, Tanzania (NIMR/HQ/R.8/Vol.IX/1604), and (3) Tanzania Commission for Science and Technology (No. 2013–273-NA-2013-101).

## Results

### Sociodemographic and obstetric characteristics

A total of 214 pregnant women participated in the study. After excluding those who missed more than 30 % of BPQ items, 211 were included for the analysis. Table 1 shows sociodemographic and obstetric characteristics of the participants. As for sociodemographic characteristics, the mean age was 27.7 (range 18–42) years, 70.1 % were married, divorced, or widowed, and 29.4 % were single. Concerning education, 84.4 % completed up to secondary school, 15.6 % completed college or university. Concerning occupation, 46.9 % were housewives and 22.7 % had jobs. For financial status, 51.7 % could use more than 5000 TSH per day, 40.3 % could use less than 5000 TSH per day. As for household assets, 71.6 % held at least one asset including house, car, or land, and 28.4 % held nothing.

**Table 1:** Sociodemographic and obstetric characteristics of the participants

	Pregnant woman (*n* = 211)	
	mean (SD)	n (%)
**Age**	27.7 (5.2)	
**Marital status**		
single		62 (29.4)
others		148 (70.1)
missing		1 ( 0.5)
**Education**		
< college		178 (84.4)
≥ college		33 (15.6)
**Occupation**		
house wife		99 (46.9)
others		48 (22.7)
missing		64 (30.3)
**Financial status**		
≤ 5000TSH		85 (40.3)
> 5000TSH		109 (51.7)
missing		17 ( 8.1)
**Household asset**		
nothing		60 (28.4)
≥ one		151 (71.6)
**Parity**		
primipara		69 (32.7)
multipara		133 (63.0)
missing		9 ( 4.3)
**Experience of caesarean section**		
no		106 (79.7)
yes		25 (18.8)
missing		2 (1.5)
**Loss of baby**		
no		106 (79.7)
yes		26 (19.5)
missing		1 (0.8)
**Distance from the nearest health center**		
≤ 1 h		134 (63.5)
> 1 h		76 (36.0)
Missing		1 ( 0.5)
**The number of antenatal care visits**		
< 4		84 (39.8)
≥ 4		119 (56.4)
missing		8 ( 3.8)
**First antenatal visit for this birth**		
≤ 3		106 (50.2)
> 3		105 (49.8)
**Decision of birthplace**		
pregnant woman alone		166 (78.7)
with/by others		39 (18.5)
missing		6 ( 2.8)
**Preference of birth attendants**		
health care provider		156 (83.9)
nonhealth care provider		55 (26.1)

Marital status: Others include married, divorced, and widowed; Occupation: Others include business, telor, teacher, specialist, farmer, assistant, media, student. Cohort was limited to those who had one or more babies for caesarian section and the experience of baby loss (*n* = 133).

For the obstetric characteristics, 32.7 % were primiparas and 63.0 % were multiparas (range 0–5). Among multiparas, 18.8 % had experience in caesarean section, and 19.5 % had experience in perinatal loss. Among all participants, 63.5 % live less than one hour from the nearest health center and 36 % live more than one hour. For ANC visits, 56.4 % attended more than 4 times and 39.8 % attended 1–3 times. As for the first antenatal visit, 50.2 % attended at first trimester and 49.8 % attended at second trimester or later. As for decision-making of birthplace, 78.7 % answered that women decided for themselves and 18.5 % answered that decision occurred with or by others. For preference of birth attendants, 83.9 % answered health care providers and 26.1 % answered family or traditional birth attendants (TBAs).

### Factors that influenced birth preparedness

Table 2 shows the result of multiple regression of the BPQ and demographic characteristics. Distance from the nearest health facility negatively influenced the total score of BPR (β=1.98, *p* = 0.01) and the factors of *BPR* (β=0.7, *p* = 0.02). Education higher than college positively influenced the total score of BPR (β = 4.76, *p* = 0.01). Decision-making of birthplace by other people (besides the women) negatively influenced *Family Support* (β=1.18, *p* = 0.03). Having jobs negatively influenced *Preparation of Money and Food* (β=-1.02, *p* < 0.01) and positively influenced the knowledge (β = 0.75, *p* = 0.03). Preference of family members and TBAs as birth attendants positively influenced *BPR* (β=-0.71, *p* = 0.03). Being single positively influenced *Preference of SBA* (β = 0.42, *p* = 0.04). Experience of losing a baby negatively influenced the knowledge (β=0.80, *p* < 0.01) and *Preference of SBA* (β=0.38, *p* = 0.02).

**Table 2:** Outcomes of multiple regression analysis of the Birth Preparedness Questionnaire

	Total of all items without K score		Total of A score		Total of B score		Total of F score		Total of H score		Total of K score		Total of M score		Total of S score		Total of W score	
	**Coefficient (95 % CI)**	***p*.value**	**Coefficient (95 % CI)**	***p*.value**	**Coefficient (95 % CI)**	***p*.value**	**Coefficient (95 % CI)**	***p*.value**	**Coefficient (95 % CI)**	***p*.value**	**Coefficient (95 % CI)**	***p*.value**	**Coefficient (95 % CI)**	***p*.value**	**Coefficient (95 % CI)**	***p*.value**	**Coefficient (95 % CI)**	***p*.value**
(Intercept)	56.53(50.92–62.13)	< 0.01**	4.52(3.15–5.89)	< 0.01**	11.71(9.7-13.72)	< 0.01**	8.24(5.08–11.39)	< 0.01**	20.5(16.06–24.94)	< 0.01**	9.9(8.07–11.74)	< 0.01**	5.07(3.7–6.45)	< 0.01**	4.07(3.02–5.11)	< 0.01**	5.02(2.82–7.23)	< 0.01**
**Age**	-0.12(-0.29-0.06)	0.19	-0.02(-0.06-0.03)	0.51	0(-0.06-0.06)	0.98	-0.01(-0.11-0.09)	0.88	-0.05(-0.19-0.1)	0.54	-0.04(-0.1-0.02)	0.24	0.01(-0.03-0.05)	0.66	-0.02(-0.05-0.01)	0.24	-0.05(-0.12-0.03)	0.21
**Marital status**																		
Others																		
Single	1.29(-0.77-3.35)	0.23	-0.13(-0.65-0.39)	0.62	0.28(-0.45-1.02)	0.45	0.46(-0.74-1.65)	0.46	1.3(-0.38-2.98)	0.14	0.39(-0.29-1.07)	0.26	0.35(-0.17-0.87)	0.19	0.42(0.02–0.81)	0.04*	-0.13(-0.97-0.7)	0.75
**Education**																		
< College																		
≥ College	4.76(1.13–8.39)	0.01*	0.89(-0.03-1.82)	0.06	0.65(-0.65-1.95)	0.33	1.57(-0.55-3.69)	0.15	-0.25(-3.23-2.73)	0.87	0.15(-1.05-1.35)	0.81	0.55(-0.37-1.47)	0.25	0.22(-0.48-0.92)	0.55	0.97(-0.51-2.46)	0.20
**Occupation**																		
House wife																		
Others	-0.31(-2.41-1.79)	0.77	0.04(-0.47-0.55)	0.87	-0.36(-1.11-0.39)	0.35	0.52(-0.64-1.68)	0.39	1.3(-0.34-2.94)	0.13	0.75(0.08–1.41)	0.03*	-1.02(-1.53–0.51)	< 0.01**	-0.09(-0.48-0.3)	0.65	0.05(-0.77-0.86)	0.91
**Financial status**																		
≤ 5000TSH																		
> 5000TSH	-0.31(-1.73-1.1)	0.67	0.13(-0.23-0.49)	0.49	0.05(-0.46-0.56)	0.85	0.76(-0.07-1.59)	0.08	-0.32(-1.49-0.84)	0.59	0.32(-0.16-0.8)	0.19	0.19(-0.17-0.55)	0.30	0.02(-0.25-0.3)	0.87	-0.33(-0.91-0.25)	0.27
**Household asset**																		
Nothing																		
≥ One	1.03(-0.78-2.85)	0.27	-0.28(-0.74-0.17)	0.23	0.28(-0.37-0.93)	0.40	0.21(-0.82-1.25)	0.69	0.42(-1.05-1.88)	0.58	-0.28(-0.9-0.34)	0.39	0.33(-0.13-0.78)	0.16	0.1(-0.24-0.45)	0.56	0.43(-0.3-1.16)	0.25
**Parity**	1.11(0.15–2.06)	0.03*	0.11(-0.14-0.35)	0.39	0.11(-0.24-0.45)	0.55	0.19(-0.38-0.75)	0.52	0.54(-0.26-1.33)	0.19	0.24(-0.09-0.56)	0.15	-0.01(-0.25-0.24)	0.94	0.06(-0.12-0.25)	0.52	0.34(-0.06-0.73)	0.10
**Experience of caesarean section**																		
No																		
Yes	-0.49(-2.4-1.43)	0.62	0.13(-0.36-0.61)	0.61	-0.13(-0.82-0.56)	0.71	0.58(-0.53-1.7)	0.31	-0.52(-2.09-1.05)	0.52	0.26(-0.37-0.9)	0.42	-0.05(-0.54-0.43)	0.84	0.27(-0.1-0.64)	0.15	-0.32(-1.11-0.46)	0.42
**Loss of baby**																		
Yes																		
No	-0.24(-1.87-1.39)	0.77	-0.27(-0.69-0.14)	0.21	0.06(-0.53-0.65)	0.84	0.18(-0.77-1.13)	0.71	0.17(-1.17-1.51)	0.80	0.80(0.25–1.34)	< 0.01**	-0.06(-0.48-0.35)	0.76	0.38(0.07–0.7)	0.02*	0.2(-0.46-0.87)	0.55
**Distance from the nearest health center**																		
> 1 h																		
≤ 1 h	1.98(0.47–3.49)	0.01*	-0.1(-0.48-0.29)	0.63	0.7(0.16–1.24)	0.02*	0.08(-0.8-0.96)	0.86	0.71(-0.53-1.95)	0.27	0.14(-0.37-0.64)	0.59	0.32(-0.07-0.7)	0.11	0.23(-0.07-0.52)	0.14	0.42(-0.2-1.03)	0.19
**The number of antenatal care visits**																		
< 4																		
≥ 4	1.26(-0.41-2.94)	0.15	-0.1(-0.51-0.31)	0.65	0.52(-0.08-1.12)	0.09	0.51(-0.44-1.45)	0.30	-0.94(-2.27-0.38)	0.17	-0.47(-1.01-0.06)	0.09	0.29(-0.12-0.7)	0.18	-0.08(-0.4-0.23)	0.60	0.2(-0.46-0.86)	0.55
**First antenatal visit for this birth**																		
> 3																		
≤ 3	-0.16(-1.58-1.26)	0.83	-0.04(-0.4-0.32)	0.83	-0.07(-0.57-0.44)	0.80	0.37(-0.46-1.19)	0.39	0.02(-1.14-1.18)	0.97	-0.05(-0.52-0.43)	0.85	0.08(-0.28-0.44)	0.66	0.23(-0.04-0.5)	0.11	0.25(-0.33-0.83)	0.40
**Decision of birthplace**																		
With/only others																		
Pregnant woman alone	0.49(-1.24-2.22)	0.58	-0.03(-0.48-0.41)	0.88	0.06(-0.56-0.68)	0.84	1.18(0.17–2.2)	0.03*	-1.17(-2.61-0.27)	0.12	-0.03(-0.61-0.55)	0.93	-0.21(-0.66-0.23)	0.36	-0.05(-0.39-0.29)	0.76	-0.01(-0.72-0.7)	0.98
**Preference of birth attendants**																		
With health care provider																		
Without health careprovider	-0.41(-2.15-1.33)	0.65	0.41 (-0.04-0.85)	0.08	-0.71(-1.33–0.08)	0.03*	-0.4(-1.41-0.62)	0.45	-0.14(-1.57-1.29)	0.85	-0.43(-1.01-0.15)	0.15	-0.19(-0.63-0.26)	0.41	-0.28(-0.62-0.05)	0.10	0.22(-0.5-0.93)	0.56

*: *P* < 0.05, **: *P* < 0.01. 95 % CI: 95 % confidence interval. *K score* Knowlede score; *H score* Home-based value score; *B score* Birth preparedness; *F score* Family support score; *A score* Avoidance of medical intervention score; *M score* Provision of money and food score; *S score* Preference of SBA score; *W score* Pregnant women’s workload score; *Marital status* Others include married, divorced, and widowed; Occupation: Others include business, telor, teacher, specialist, farmer, assistant, media, student.

### Scores of BPQ

Table 3 shows the total score and scores of each factor in BPQ. The mean score of knowledge was 9.47 (SD0.92), with a maximum score of 10. *Provision of money and food* was 5.75 (SD0.62), with a maximum score of 6. Compared with the data from pregnant women in rural areas, those two scores were close to the maximum scores.

**Table 3:** Comparison of BPQ data in urban and rural areas

	Urban (*N* = 211)	Rural (*N* = 42)^a^	
	mean (SD)	mean (SD)	Range
**Knowledge**	9.47 (0.92)	7.85 (1.87)	0–10
**Total BPR**	58.08 (2.80)	60.75 (4.70)	24–72
**Home-based value**	19.91 (1.73)	17.32 (3.49)	7–21
**Birth preparedness**	12.42 (0.97)	14.13 (1.07)	5–15
**Family support**	10.62 (1.73)	10.02 (1.76)	4–12
**Avoidance of medical intervention**	4.06 (0.58)	3.92 (0.61)	2–6
**Provision of money and food**	5.75 (0.62)	4.77 (1.28)	2–6
**Preference of SBA**	4.00 (0.45)	5.78 (0.70)	2–6
**Pregnant women’s workload**	5.16 (1.16)	4.80 (1.31)	2–6

^a^From Shimpuku et al., 2018

## Discussion

This study illustrated pregnant women’s BPR and the factors that influenced it in the city area of Tanzania. From the results of multiple regression analyses, some findings were congruent with the findings of previous studies, and some were not. These might have been influenced by the unique developmental stage of the city area of Tanzania, which recently became a middle-income country. Although ANC more than four times [21–23], first ANC within the first trimester [22], income [24], and age [24–26].showed significant relationships with BPR in previous studies, they were not found to be associated in this study.

Our finding suggested that when birthplace was decided by others besides the women themselves, women had less family support. This could mean that when decision-making power within the household belonged to other family members, women’s preferences or needs were not heard. A study in rural Tanzania showed perceptional gaps among women, husbands, and other family members about intention for birthplace and identified the needs to promote communication among family members [27]. The current study showed similar tendency even in the city area and the needs of education for the family. A study in Nepal supported that joint decision-making with the husband showed better preparedness [28]. Another research of a qualitative synthesis in 19 countries including both high-income and low/middle-income countries revealed strong influence of familial and sociocultural norms on decision-making; saying that women usually wish to retain a sense of personal achievement and control by being involved in decision-making [29]. Also, they explained that beliefs about what matters to women are influenced by familial experiences, local cultural norms, and values as women interpreted their expectations of what could and should happen through the lens of family birth stories, and cultural and social norms. Therefore, involvement of pregnant women in decision-making of birthplace is important, and it should be supported by their family and society.

It was also a surprising but important finding that being single had positive influence on preparation of money and food as well as preference of SBA. Several studies showed opposite results that being single had negative influence on preparedness [30–32]. For example, a study in Ethiopia revealed that being single was one of the significant predictors of unplanned home delivery [33]. In contract, a study in rural Masaai community in Kenya showed that currently not married is positively associated with health facility deliveries [34]. It is possible that being single mean being in poverty and therefore it is difficult to prepare or reach a health facility in rural areas. However, being single mean that women could decide by themselves. In the urban population, it is plausible that being single does not necessarily equal to being poor, and positively influences BPR.

It was a surprising finding in this study that having jobs negatively affected preparation of money and food. As women having jobs were found to have better knowledge, lower preparedness was not due to lack of knowledge. It is plausible that women who have jobs are likely to be busy with having multiple roles, which could negatively affect their preparedness. In a study of family caregivers of people with schizophrenia in Mauritius, caregivers had poor emotional well-being, poor physical and social health, and an increase in financial worry when their roles increased [35]. Findings from former studies were incongruent and interpreted having jobs as higher economic status that is positively related to preparedness. For example, a study in Ethiopia showed that occupational status of mother (government or NGO) had positive influence on BPR compared to others (student or daily laborer) [36]. Another study in Ethiopia stated that having occupation (government employee or merchant) and being in the higher wealth quintile were variables positively associated with BPR and complication readiness [37]. A study in India also connected women’s occupation with higher socioeconomic status and those who were categorized as laborer, cultivation, and service had better outcomes on BPR [38]. Lenga et al. [39] showed relationships between socioeconomic status and use of maternal health services and stated that the utilization of skilled birth assistance between women in poorer wealth quintile and women in richer household wealth quintile were significantly wider in rural areas than in urban areas. Therefore, it is plausible that the influence of socioeconomic status was less in the urban area populations; rather, having multiple roles could strongly influence BPR.

It was another surprising finding that preference of family members or TBAs, not SBAs, had positive influence on BPR. The opposite is usually considered the case; preference of SBAs could lead to better preparation so that they could give birth with SBAs. For example, a study in Ethiopia showed that those who were counseled to identify SBAs had better preparedness [25]. The result of the current study could be interpreted to mean that women, in the urban area, had access to information and learned about positive influences of having familiar labor companion on their birth experience. In high-income countries, births are often attended by family members or a doula. A Cochrane review of 51 articles described that labor companions supported women in four different ways. They provide informational support, act as advocates, provide practical and emotional support, and they helped women to have a positive birth experience [40]. Although studies on women’s perceptions of birth experience in Tanzania are limited, Shimpuku et al. [5] described the importance of caretaking role of the family during childbirth and suggested family attendance at birth, based on women’s perceptions in the study in rural Tanzania. The WHO also published the recommendations on positive pregnancy experience that include maintaining women’s physical and sociocultural normality, maintaining a healthy pregnancy for both mother and baby (i.e. preventing and treating risk factors, illness and death), providing an effective transition to positive labor and birth, and assisting women in achieving positive motherhood (i.e. maternal self-esteem, competence, and autonomy) [41]. As this study shows that preference of family or TBAs could positively influence BPR, supporting family involvement toward women’s positive experience, a global trend, should be applied as women wish.

Another important finding is that experience in losing a baby had negative influence on knowledge and preference of SBA. The opposite is generally considered; some previous studies showed that women who had experience of miscarriage had higher BPR [42–44]. The finding of this study could mean that women in urban Tanzania did not have opportunity to learn about BPR for this pregnancy after losing a baby. Such learning opportunity should be promoted, and to this end, it is important to know how women perceive the death of a baby. In a study in mountain villages of Nepal [45], women had a strong belief in religio-cultural determinants of perinatal death, which demonstrates that medical interventions alone were not sufficient to prevent these deaths, but broader social determinants, which are highly significant in local life. It is important to consider the ways to convey the message of BPR so that it will be culturally acceptable and understood by women who have lost a baby.

Another finding suggests that living far from the nearest health center negatively influenced BPR. It is plausible that women may experience difficulties in reaching a health facility and think of giving birth at home. Other studies in Africa also supported negative association between BPR and distance from health facility [6, 9, 34, 46]. However, it does not necessarily mean that we should give up on women who live far from the health facility. A study in Thailand, where MMR is lower and facility birth rate is higher than those in low-income countries, distance from health facility had opposite effect. The distance from health facility positively affected BPR [47]. It is considered that if it is the social norm to give birth at a health facility, women become understandably worried if their house is far from the health facility, and thus prepare well. This could indicate the potentials of educating women and community especially in the city area where access to a health facility is feasible if they prepare well. Women can be taught that they need to prepare well when they live far from a health facility.

The information from this study can give important insights for development of a smartphone app. First, the solution of mHealth can benefit pregnant women in the study population as the information can reach those who live far from health facilities, and the information can be shared with the family members when they are key decision-makers within the household. As women in the city area have more professional demanding jobs, information needs to be quickly accessed and understood. They might agree with positive experience; the contents of the app should include information from the WHO recommendations on women’s positive experience during pregnancy and childbirth. The contents should address women from diverse background including single women and women who lost their baby at previous pregnancy.

## Limitations

This study was significant as there is not much former research available in terms of pregnant women’s BPR in the city area of Tanzania. The information can be used for development of a smartphone app. However, generalization of the study needs careful consideration as the study used convenient sample. The next larger study we plan should use more rigorous sampling methods and larger sample size.

As a measurement of BPR, knowledge items could be changed according to the context. As the scores were almost the maximum, which means women in the urban area have this knowledge already, it could cause ceiling effect and not properly evaluate the effect of a study.

## Conclusions

The findings showed an updated information on pregnant Tanzanian women living in an urban area where rapid environmental development was observed. Women who resided far from the health facilities, whose birthplace decision-making was taken by others besides the women, those having jobs, and those who had experienced the loss of a baby, were negatively affected in terms of BPR. Our future study that will apply a smartphone app intervention will use this information for its contents and guidance of analysis. Although generalization of the study needs careful consideration, it is important to reconsider issues surrounding BPR as women’s roles both in the family and society are more, especially in urban settings.

## Data Availability

The datasets used and analyzed during the current study are available from the corresponding author upon reasonable request.

## Abbreviations

TBAs: Traditional Birth Attendants
SBAs: Skilled Birth Attendants
BPQ: Birth Preparedness Questionnaire
BPR: Birth Preparedness
WHO: World Health Organization
MMR: Maternal Mortality Ratio
DHS: Demographic and Health Survey
ANC: Antenatal Care
AMO: Assistant Medical Officer
TSH: Tanzanian Shillings
SD: Standard Deviation

## Declarations

### Authors’ information

YS is a Japanese midwifery researcher who obtained her PhD from the University of Illinois at Chicago, USA. She has been conducting research on maternal child health and midwifery in Tanzania since 2008. She speaks fluent English and Kiswahili which were necessary for conducting this study. KI is also a Japanese midwife and obtained a master’s degree under the supervision of YS. She had 3-year volunteer experience in Tanzania and is fluence in Kiswahili. NH is a Japanese nurse and a data scientist who was involved in the study as a PhD student of YS. KK is a Japanese nurse scientist who is specializing in statistics. BM and DM are Tanzanian midwifery researchers who provided cultural understanding and language translation for the data collection.
